# Investigation of Adsorption Kinetics and Isotherms of Synthetic Dyes on Biochar Derived from Post-Coagulation Sludge

**DOI:** 10.3390/ijms26167912

**Published:** 2025-08-16

**Authors:** Barbara Pieczykolan

**Affiliations:** Faculty of Energy and Environmental Engineering, Silesian University of Technology, Konarskiego 18, 44-100 Gliwice, Poland; barbara.pieczykolan@polsl.pl; Tel.: +48-32-237-28-21

**Keywords:** biochar, post-coagulation sludge, dye adsorption, adsorption kinetics, adsorption isotherm

## Abstract

An activated biochar was produced from post-coagulation sludge (also called water treatment residuals or water treatment sludge) in the pyrolysis process at 800 °C in a nitrogen atmosphere and chemical activation using NaOH. The produced adsorption material was characterised by an S_BET_ surface area of 439 m^2^/g, a total volume of pores of 0.301 cm^3^/g, and an average pore size of 1.4 nm. FTIR analysis reveals the presence of primarily C-H, C-O, N-H, C-N, and O-H groups on the activated biochar surface. The batch adsorption process was conducted for three dyes: Acid Red 18, Acid Green 16, and Reactive Blue 81. In the study, the effect of pH, contact time, adsorption kinetics, and adsorption isotherm was determined. The studies showed that, for all dyes, the highest efficiency of the process was achieved at a pH of 2. The results indicate the occurrence of a chemical adsorption process, as evidenced by the best fit to the experimental results obtained with the pseudo-second-order kinetics model and the Elovich model. In the case of the adsorption isotherm, the SIPS model best describes the adsorption for Acid Red 18 and Reactive Blue 81, and the Jovanovic model describes the adsorption of Acid Green 16.

## 1. Introduction

One of the unit processes commonly used for water purification is the coagulation/flocculation process. Its main task is to remove colloidal, organic, and fine suspensions from water. It involves introducing appropriate reagents to the purified water, whose task is to destabilise the colloidal system (neutralise or significantly reduce the zeta potential) and enable the generation of a flocculated suspension. The floccules produced in the process have sorption properties, into which the pollutants removed from the water are incorporated [1,2,3].

The most commonly used reagents for the coagulation/flocculation process are aluminium or iron salts, such as FeCl_3_, Al_2_(SO_4_)_3_, and Fe_2_(SO_4_)_3_, but also pre-hydrolysed compounds such as: polyaluminium chloride, polyhydroxide, aluminium chloride, hydroxide, etc. [1,2].

The generated floc suspension contains aluminium or iron hydroxides in its structure. Due to the pollutants removed in the water purification process and the individual characteristics of the purified water, the physicochemical composition of the generated sediments can be very different. Surface waters are characterised by the presence of compounds causing colour and turbidity, as well as a variable content of dissolved organic and inorganic compounds, as well as microorganisms (bacteria, algae, and higher plants). Water will contain, among others, such compounds as cations and anions, humic substances, nitrogen compounds, phosphorus compounds (organic and inorganic), polycyclic aromatic hydrocarbons, pesticides, etc. [2,4,5,6].

During the coagulation and flocculation process, most of the contaminants mentioned above are removed, with the effectiveness of their removal varying depending on the type of contaminant. This will result in the presence of these contaminants in the structure of the post-coagulation sludge [2,4,5]. The post-coagulation sludge is a two-phase mixture consisting of solid-phase floccules and liquid (water). The sludge hydration is typically above 99% (for sludge not subjected to any thickening processes) or around 95% (for thickened sludge). Alum sludge (post-coagulation sludge formed during coagulation/flocculation of water using aluminium salts) is characterised by various contents of organic compounds (COD and BOD_5_) as well as the content of total suspended solids and heavy metals. Its composition depends primarily on the quality of the purified water [1,7].

Sludge produced during water purification by the coagulation/flocculation process is usually stored in landfills, usually pre-thickened or dewatered (before which it is subjected to various conditioning methods). However, research is already underway on other methods of management and disposal of these sludges. Coagulants can be recovered from sludge [8], and sludge can be used as a filter material [1], but also as an adsorption material for removing pollutants [9,10] or soil remediation [11]. Additionally, post-coagulation sludge, and particularly alum sludge, can be disposed of as a component of building materials [7] or cement [12,13].

The research results presented in this publication describe the adsorption process of three types of synthetic dyes, Acid Red 18 (AR 18), Acid Green 16 (AG 16), and Reactive Blue 81 (RB 81). AR 18 and RB 81 dyes belong to the group of azo dyes, whereas AG 16 belongs to the group of triphenylmethane dyes. These dyes are used in various industries, including dyeing wool, silk, and cotton fabrics, as well as polyamide; and for printing, plastic, wood, medicine, and cosmetics [14,15,16]. However, it should be remembered that they can pose a threat to both the natural environment and the health and life of animals and humans.

Triphenylmethane dyes, such as Acid Green 16 (AG 16), malachite green (MG), crystal violet (CV), and brilliant green (BG), exhibit significant biological and environmental toxicity [17]. AG 16 was confirmed to be genotoxic in mice, as it causes chromosome damage manifested by the formation of micronuclei [18,19]. MG causes hematological and biochemical changes in fish and binds to hemoglobin, suggesting multi-organ toxicity [20]. CV can irritate the eyes and skin and have mutagenic and carcinogenic properties. BG penetrates the skin and can migrate from packaging into food [20]. Triphenylmethane dyes are poorly biodegradable, and accumulate in water and organisms, and their metabolites (e.g., leuco-MG) can be carcinogenic and teratogenic [21]. In addition, this type of dye may pose a risk to human health. Studies conducted on the Brilliant Blue dye have shown that it can cause anaphylactic reactions and other hypersensitivity symptoms [22].

Azo dyes, on the other hand, can be decomposed in the human body, especially in the intestines, under the influence of bacterial microflora, into aromatic amines, which have been proven to be carcinogenic [23]. Studies conducted by Jiang et al. [24] on *Danio rerio* fish embryos have shown that azo dyes (such as tartrazine or Allura Red) can cause cardiac edema and spinal deformities. Azo dyes have also been shown to damage the DNA of soil bacteria, which negatively affects soil fertility [25]. In the case of the impact of azo compounds on human health, studies indicate that there is a link between benzidine-based compounds and the occurrence of bladder cancer in textile workers [26]. Additionally, some dyes may be contaminated with carcinogens, which negatively affect the intestinal microflora and may cause hyperactivity in children [27]. The dye AR18 used in the studies described in this article exhibits potential genotoxic effects due to its ability to damage DNA. As a result, long-term or excessive intake may be harmful to human health, with possible carcinogenic effects. Moreover, increased exposure to AR18 has been associated with allergies, hyperactivity in children, and asthma, leading the EFSA to establish an acceptable daily intake of less than 0.7 mg/kg body weight [28,29].

Reactive dyes exhibit significant toxicity to the environment, animals, and humans. In the case of reactive dyes, studies show that compounds such as Reactive Red 239 are highly toxic to aquatic organisms, including the bacteria *Vibrio fischeri*, the crustacean *Daphnia similis*, and the embryos of the snail *Biomphalaria glabrata* [30]. Additionally, the dyes Reactive Blue 15 and Reactive Orange 16 induce deformations and delays in the development of *Danio rerio* fish embryos [31]. In Ames tests, Reactive Blue 19 dye exhibits mutagenicity in the presence of metabolic activation (S9) [32], and Reactive Green 19 causes DNA damage in human skin models [33]. In humans, reactive dyes can act as haptens, inducing IgE-mediated reactions and symptoms of occupational asthma in textile industry workers [34]. These dyes are difficult to biodegrade, accumulate in the aquatic environment, and may exhibit mutagenic and cytotoxic effects [34].

The publication presents research on the possibility of producing biochar from sludge generated during water purification using the coagulation/flocculation process. The activated biochar produced in the pyrolysis and chemical activation process was used for the batch adsorption of three synthetic dyes, AR 18, AG 16, and RB 81.

The activated biochar produced from both alum sludge and sewage sludge displays a high porosity and a large BET surface area, and contains functional chemical groups, making it an effective adsorbent for various pollutants, including phosphates, heavy metals, dyes, and pharmaceuticals [35,36,37]. The near-complete removal of NH_3_-N, NO_2_-N, and PO_4_-P has been demonstrated using a mixture of biochar and alum sludge [35]. In the case of sewage sludge, this biochar effectively removes organic pollutants and pharmaceuticals with an efficiency often exceeding 90% [36]. Additionally, biochar can be regenerated and reused, which reduces waste, supports a circular economy model, and helps limit CO_2_ emissions [37]. The production of biochar from waste materials, such as sewage sludge, alum sludge, or post-coagulation sludge, provides an effective method for removing pollutants while also serving as a sustainable strategy for managing challenging waste streams. This approach reduces the amount of waste that needs to be disposed of, minimises landfill use, and recovers valuable resources in the form of functional adsorbents. Therefore, converting waste into biochar not only aids in environmental protection but also enhances the overall sustainability of wastewater treatment processes.

## 2. Results and Discussion

### 2.1. Activated Biochar Characteristics

#### 2.1.1. FTIR Measurements

**Figure 1:**
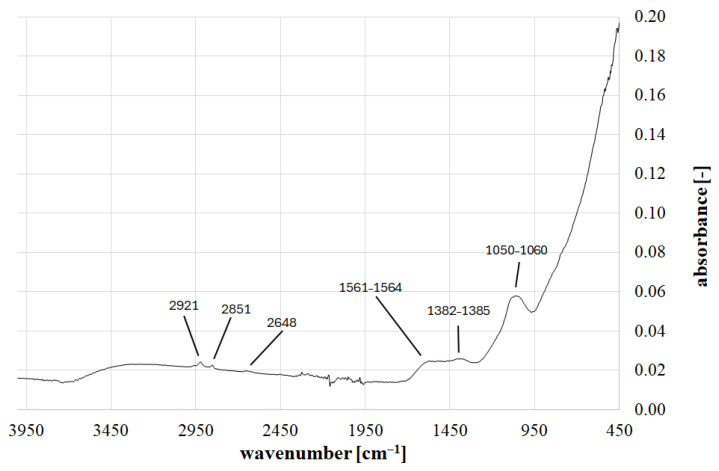
FTIR spectrum for biochar.

FTIR analysis revealed the presence of broad absorption bands, primarily in three wavelength ranges: 1050–1060 cm^−1^, 1382–1385 cm^−1^, and 1561–1564 cm^−1^. Sharp absorption peaks were measured at wavelengths of 2648 cm^−1^, 2851 cm^−1^, and 2921 cm^−1^. The most intense measurement, where the most significant local increase in absorption value was recorded, was observed in the 1050–1060 cm^−1^ band (Figure 1).

The broad band in the 1050–1060 cm^−1^ range indicates the presence of C-O or -C-O-C- groups. The absorption band in the 1382–1385 cm^−1^ range indicates the presence of C-H bending groups, while the broad absorption peak in the 1561–1564 cm^−1^ range corresponds to N-H bending and C-N stretching groups. The peak at 2851 cm^−1^ indicates the symmetric stretching of C-H in -CH_2_- groups. The presence of a peak at 2648 cm^−1^ corresponds to C-H stretching vibration, and at 2921 cm^−1^ to asymmetric C-H stretching vibration [38].

#### 2.1.2. Specific Surface Area Measurement

The measurement of the specific surface area, pore size, and pore volume using the BET adsorption method revealed that the prepared activated biochar had a surface area of 439 m^2^/g. Moreover, the total volume of pores < 50 nm was 0.301 cm^3^/g, and the pore volume determined by the Quenched Solid Density Functional Theory (QSDFT) method was 0.279 cm^3^/g. The average size of pores < 50 nm (slit shape) was 1.4 nm, and the pore surface area using the QSDFT method was 400 m^2^/g. Moreover, the dominant pore size determined by the QSDFT method (slit shape) was 0.85 nm.

The measured specific surface area of the activated biochar is similar to that of macadamia-nut-residue-derived biochar (398.1 m^2^/g [39]) and higher than that of biochar produced from protein-modified rice husk (189.22 m^2^/g [40]), activated carbon derived from the stems of *Phyllanthus reticulatus* (124.7 m^2^/g [41]), and tilapia bone-based biochar (124 m^2^/g [42]). In the case of biochar produced from pine tree logging residues, the specific surface area was several times higher, ranging from 2524 m^2^/g to 3141 m^2^/g, depending on the method of adsorbent production [43].

#### 2.1.3. Microscopic Images

**Figure 2:**
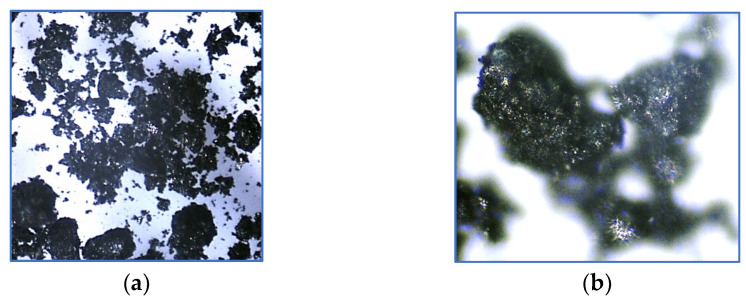
The microscopic image of biochar at (**a**) 40× and (**b**) 100× magnification.

The produced activated biochar is characterised by a relatively diverse grain size (Figure 2). The microscopic images taken (magnification 40× and 100×) indicate the occurrence of a non-homogeneous, corrugated, and differentiated surface structure.

### 2.2. The Effect of pH

**Figure 3:**
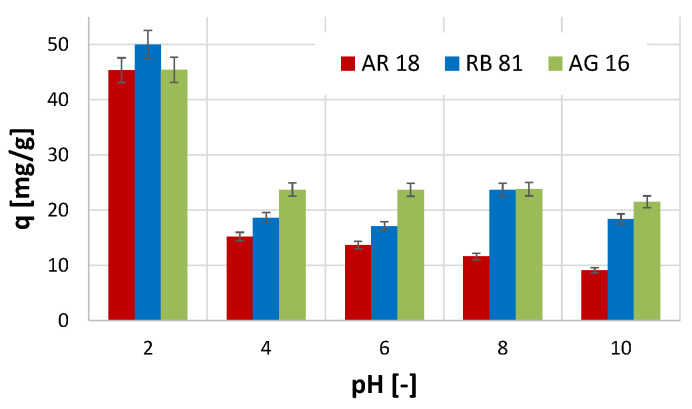
The effect of pH on the efficiency of the adsorption process.

The conducted studies on the effect of pH on the efficiency of the adsorption process showed that, in the case of all three dyes tested, the highest efficiency of the process was noted at pH 2 (Figure 3). At these conditions (pH 2), the highest amount of the adsorbed dye per unit mass of the activated biochar was obtained for the RB 81 (*q* = 50 mg/g). In comparison, in the case of the other two dyes, the amount of the adsorbed dye was smaller and equalled 45 mg/g. Increasing the pH value of the reaction conditions resulted in a decrease in the efficiency of the adsorption process efficiency.

The most significant decrease in efficiency was observed for the AR 18 dye, where, with an increase in pH from 4 to 10, the amount of adsorbed dye gradually decreased. In the case of the AG 16, a decrease in the efficiency of removing this dye was also noted with an increase in the pH value. However, in this case, the amount of adsorbed dye did not change significantly with an increase in pH value in the range of 4 to 10.

The observed phenomenon may indicate that a negative charge characterises the surface of the activated biochar, and only strongly acidic reaction conditions contribute to a change in the surface charge to a positive one (excess of H^+^ ions in the solution). The tested dyes in the hydrolysis process acquire a negative charge (and, therefore, become anions), and, as a result of the protonation phenomenon of the activated biochar surface, they can be attracted to its surface [44]. Thus, in a pH environment higher than 2, the absorption efficiency decreases significantly. A study conducted by Kavitha et al. [41], which utilised an adsorbent prepared from *Phyllanthus reticulatus* for the adsorption of Reactive Orange 16, also demonstrated that the highest adsorption efficiency was achieved at pH 2. Moreover, in the study described by Khasri et al. [45] on the removal of Remazol Brilliant Violet 5R using melunak- and rubberwood-sawdust-based activated carbon, as well as in the study on the adsorption of Direct Red 5B, Direct Black 22, Direct Black 71, and Reactive Black 5 using spent mushroom waste described in the work of Alhujaily et al. [46], the most favourable pH value for adsorption was 2.

### 2.3. The Effect of Contact Time on Adsorption Efficiency

**Figure 4:**
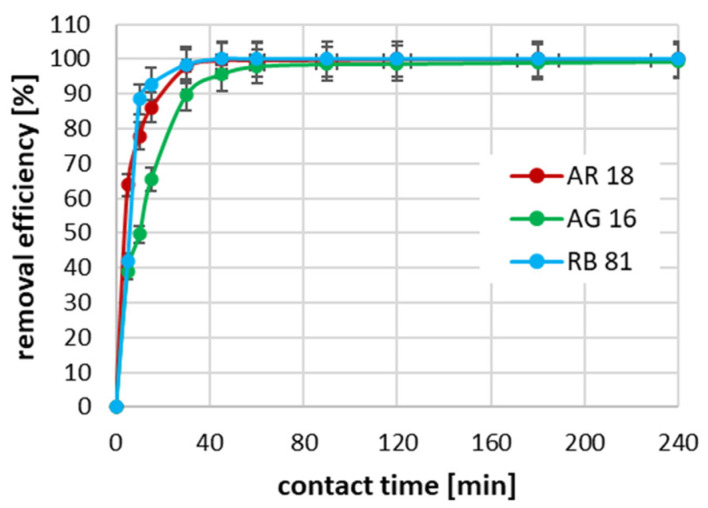
The impact of contact time on adsorption effectiveness for an initial concentration of 100 mg/dm^3^.

The conducted studies on the effect of the contact time between the dye and the activated biochar surface on the adsorption efficiency showed that, for all tested dyes at an initial concentration of 100 mg/dm^3^, the process was the fastest in the first 60 min (Figure 4). It can be seen that, in the case of the RB 81, the degree of dye removal was almost 90% in the first 10 min of reaction. In the case of AR 18, the 10 min contact time allowed for dye removal resulted in a reduction of almost 80%. In contrast, for AG 16, after 10 min of adsorption, only about 50% of the dye was removed. In the case of the RB 81 with an initial concentration of 100 mg/dm^3^, the complete removal of this dye was achieved after 45 min of absorption; in the case of the AR 18, 100% adsorption efficiency was achieved after 120 min; while, in the case of the AG 16, the complete removal of the dye was not achieved during the tests, i.e., until the 240th minute of the process.

**Figure 5:**
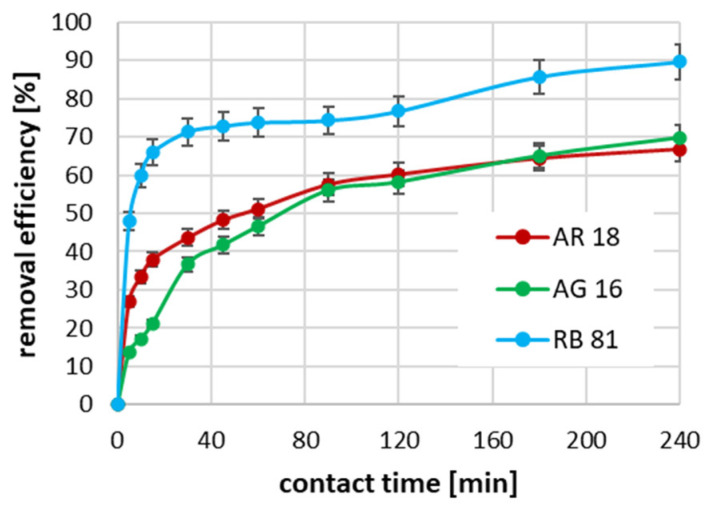
The impact of contact time on adsorption effectiveness for an initial concentration of 700 mg/dm^3^.

In the case of using an initial concentration of dyes equal to 700 mg/dm^3^, the highest process rate was again observed for RB 81 (Figure 5). In this case, after 30 min of adsorption, 71% of the dye was removed from the solution, and extending the contact time up to 240 min contributed to an increase in efficiency up to 89.5%. In the case of AR 18 and AG 16, with an initial concentration of 700 mg/dm^3^, a similar rate of change in the increase in efficiency of removing these dyes was observed. The highest growth was also observed in the first 30 min of adsorption. However, 43.6% and 35.4% of AR 18 and AG 16 removal were obtained, respectively. Increasing the duration of the process contributed to an increase in the adsorption efficiency of both dyes to approximately 68%.

A similar phenomenon, where an increase in the initial concentration of the pollutant to be removed required longer contact times to achieve equilibrium, and the appropriate process efficiency, was noted in the studies described by Khasri et al. on the removal of the Remazol Brilliant Violet 5R dye [45]. In the studies conducted by Alhujaily et al. [46], the impact of the initial concentrations of Direct Red 5B, Direct Black 71, Direct Black 22, and Reactive Black 5 dyes on the time required to reach equilibrium was evident.

### 2.4. The Results of Adsorption Kinetics

The results from experiments evaluating the effect of the contact time on the adsorption efficiency were utilised to determine the adsorption kinetics. Nonlinear estimation, based on the minimisation of Root Mean Square Error (*RMSE)*, was employed to identify the parameters of kinetic models, the formulae for which are presented in Table 1. For the intraparticle diffusion model, linear estimation was applied.

The conducted analysis of adsorption kinetics showed that, in the case of the AR 18, the best matches to the experimental data were obtained for the pseudo-second-order and Elovich models (Table 2). The highest *R*^2^ values were measured for both the calculated and experimental data. This indicates that the adsorption process of the dyes used in these studies showed a more chemical than physical adsorption character. In the case of the AG 16 and RB 81, depending on the initial concentration value, the best model matches were obtained for the pseudo-first-order model when *C*_0_ = 100 mg/dm^3^. When the initial concentration of these dyes was 700 mg/dm^3^, the best matches were obtained for the pseudo-second-order and Elovich models. These results suggest that, depending on the number of molecules in the solutions that contact the activated biochar surface, the nature of the adsorption process may vary. Both the pseudo-second-order model and the Elovich kinetics model describe the chemical adsorption process [53,54,55,56,57]. Furthermore, the Elovich model represents the type of adsorption where the increase in the amount of adsorbate is followed by an exponential decrease in the adsorption rate [57,58].

The results of the study also indicate that, for an initial concentration of 100 mg/dm^3^, the highest adsorption constant rate values were obtained for AR 18, followed by RB 81, and the lowest for AG 16. However, in the case of using a higher initial concentration of all dyes (*C*_0_ = 700 mg/dm^3^), a significantly higher adsorption constant rate was observed for the RB 81 dye than for the other two tested dyes.

Moreover, in the case of all three dyes, an increase in the initial concentration from 100 mg/dm^3^ to 700 mg/dm^3^ resulted in a decrease in the value of the adsorption constant rate. This phenomenon results from the fact that, with an increase in the number of dye molecules present in solutions, there is a greater competition of these molecules for free active sites on the activated biochar surface. This results in a decrease in the adsorption rate and, consequently, in obtaining lower values of the rate constants *k*_1_ and *k*_2_ [59].

**Figure 6:**
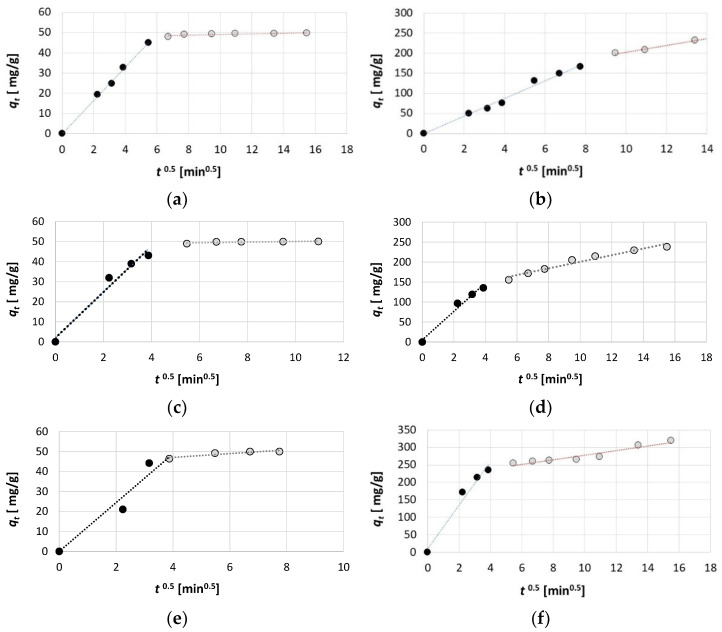
The intraparticle diffusion model graphs for (**a**) AG 16 100 mg/dm^3^, (**b**) AG 16 700 mg/dm^3^, (**c**) AR 18 100 mg/dm^3^, (**d**) AR 18 700 mg/dm^3^, (**e**) RB 81 100 mg/dm^3^, and (**f**) RB 81 700 mg/dm^3^.

An analysis of the results of the intraparticle diffusion model allows us to determine the adsorption mechanism. Based on the *q_t_* = f(*t*^0.5^) graph for all three dyes and both initial dye concentrations, it can be observed that the curve divides into two stages with different adsorption rates (Figure 6). The first stage of the graph is steeper and represents the film diffusion process stage. At this stage, dye molecules migrate from the interior of the solution to the surface of the activated biochar, and this stage is also called outer diffusion [60,61,62]. The second stage, which is already characterised by a significantly lower slope, illustrates the inner diffusion process (also known as intraparticle diffusion), where molecules move from the external surface of the adsorbent to the interior of the pores [60,63].

The values of the parameters of the intraparticle diffusion model determined by linear estimation indicate that, for all three dyes tested, as well as for both initial concentrations, the intraparticle diffusion rate constant (*K_IPD_*) is much higher in the first stage (outer diffusion) than in the second (inner diffusion) (Table 3). This indicates that the adsorption process during the transfer of particles from the interior of the solution to the surface of the activated biochar is significantly faster than the subsequent adsorption that occurs already inside the pores. It is also noticeable that, for each dye, the intraparticle diffusion rate constant is much higher when a higher initial concentration of the dye (700 mg/dm^3^) is used. Moreover, the highest values of the *K_IPD_* were obtained for RB 81, and the lowest for AG 16. This confirms that, in the case of RB 81, the process of retaining dye molecules on the surface of the activated biochar was the fastest, and the equilibrium state was reached the most rapidly.

In the intraparticle diffusion model, the parameter *C* determines the degree of influence of the boundary layer on the adsorption process. The higher the value of this parameter, the more the boundary layer limits the rate of the external mass transfer process, which is related to the occurrence of a greater thickness of the boundary layer [64,65,66]. In all cases, in the inner diffusion stage, the value of the parameter *C* was greater than 0 (Table 3). Moreover, it should be noted that significantly higher values of *C* were calculated for the higher initial concentrations of dyes used (for 700 mg/dm^3^). This is due to the fact that, at a higher concentration, a more pronounced effect of the number of molecules migrating near the activated biochar surface was visible, resulting in a thicker boundary layer than in the case of a lower concentration (100 mg/dm^3^).

An analysis of the obtained data and graphs from the intraparticle diffusion model indicates a similar duration of inner diffusion for the RB 81 and AR 18 for both initial dye concentrations (Figure 6). In this case, the first phase lasted up to 15 min of the adsorption process and was much shorter than the second phase, which lasted up to 120 min for AR 18 and *C*_0_ = 100 mg/dm^3^, up to 60 min for RB 81 and *C*_0_ = 100 mg/dm^3^, and up to 240 min for the concentration of *C*_0_ = 700 mg/dm^3^ of both dyes. In the case of AG 16, the outer diffusion lasted up to 45 min for *C*_0_ = 100 mg/dm^3^ and up to 60 min for *C*_0_ = 700 mg/dm^3^. This indicates that, in the cases of RB 81 and AR 18, the inner diffusion process played the most significant role in determining the adsorption process rate. In contrast, for AG 16, the outer diffusion process also had a substantial influence on the adsorption process rate.

### 2.5. The Results of Adsorption Isotherm

Based on the experimental results related to the determination of the adsorption isotherm, the parameters of the selected isotherm models, whose equations are given in the Table 4, were determined using nonlinear estimation (by minimising *RMSE* value), and the *R*^2^ coefficient value was calculated as a measure of the strength of the model fit to the experimental results.

The analysis revealed that, for AR 18 and RB 81, the highest degree of fit between the model results and the experimental results was achieved for the SIPS isotherm, with values of 0.971 and 0.952 for AR 18 and RB 81, respectively (Table 5). On the other hand, for AG 16, the highest *R*^2^ value was measured for the Jovanovic isotherm (0.994), and it was slightly lower for the SIPS model (0.993). The Jovanovic isotherm describes localised monolayer adsorption without lateral interaction. However, unlike the Langmuir isotherm [74,75], it assumes the possibility of mechanical interactions between the adsorbate molecules and the activated biochar surface [76,77]. In contrast, the SIPS isotherm model is a combination of the Freundlich and Langmuir models, which describe adsorption on a heterogeneous surface, thereby avoiding the limitations associated with increased adsorbate concentration [78].

The values of the determined parameters of the individual isotherm models allow conclusions to be drawn regarding the value of the maximum monolayer adsorption capacity, the degree of heterogeneity of the activated biochar surface, the affinity of the adsorbate and adsorbent, the mean energy of adsorption, and the type of adsorption (physical or chemical).

Analysing the values of *q_m_* (in the Langmuir model [74,75]), *q_max_* (in the Jovanovic model [79]), and *Q_s_* (in the D–R model [80]), it can be seen that the highest adsorption capacity values were obtained for RB 81 (Table 5). However, in the case of AR 18 and AG 16, these values are similar. Moreover, the *K_F_* parameter is related to the adsorption capacity [74], and the values obtained from the estimation also indicate that biochar showed the highest sorption capacity values for AR 18 and RB 81, and the lowest for AG 16.

The 1/*n* parameter in the Freundlich model is related to the affinity of the adsorbate to the surface of activated biochar [74]. In this case, the highest value was measured for AG 16 and the lowest for AR 18 (Table 5). This parameter also determines the heterogeneity of the adsorbent surface [81,82,83], similarly to the value of the *SP* parameter in the SIPS model [84]. In both cases, the lower the value of these parameters, the greater the surface heterogeneity. In general, the results indicate a quite significant surface heterogeneity (almost all values are significantly lower by 1 and closer to 0). The constants *K_L_*, *K_J_*, and *K_S_* in the Langmuir, Jovanovic, and SIPS models, respectively, are related to the energy of adsorption [79,84]. The research results indicate that the activated biochar-AR 18 and activated biochar-RB 81 systems exhibited higher adsorption energy compared to the activated biochar-AG 16 system. Moreover, the determined mean free energy *E* value from the D–R model also indicates that the lowest value was calculated for AG 16. However, all obtained E values indicate physical adsorption, not chemical adsorption [85]. Furthermore, according to the Temkin isotherm model, the *B_T_* parameter values related to the heat of adsorption were determined, revealing that this parameter is positive for all three dyes. This indicates that an exothermic process occurs [53,86]. Notably, the highest *B_T_* value was observed for the adsorption of AG 16, while the lowest was recorded for RB 81.

The activated biochar produced from post-coagulation sludge and used for adsorption of AR 18, AG 16, and RB 81 was characterised by an adsorption capacity determined from the Langmuir model at the level of 202.07 mg/g (AR 18), 204.49 mg/g (AG 16), and 253.54 mg/g (RB 81). Similar values of sorption capacity were obtained in the studies described by Khasri et al. [45], where biochar produced from melunak and rubberwood sawdust was used for the adsorption of Remazol Brilliant Violet 5R dye (Table 6). Slightly lower adsorption capacities were obtained for activated carbon derived from the stems of *Phyllanthus reticulatus* during the adsorption of Reactive Orange 16 [41]. However, in the case of studies conducted using Macadamia-nut-residue-derived biochar for adsorption of β-naphthol and Reactive Black 5 [39], as well as using protein-modified rice husk biochar for the adsorption of New Coccine azo dye [40], the sorption capacity values were much lower (at the level of several to a dozen or so mg/g). In contrast, experiments conducted by Grimm et al. [43], where activated carbon was produced from pine tree logging residues and used to remove Reactive Black 5, showed a significantly higher adsorption capacity, ranging from 1260 to 1363 mg/g.

## 3. Materials and Methods

### 3.1. Activated Biochar Preparation

In the study, the sludge produced during the purification of surface water using the coagulation/flocculation process in the municipal drinking water treatment plant was transformed into activated biochar in two stages. In the first step, the dehydrated, dried, and crushed sludge was subjected to pyrolysis in a quartz tube furnace (PRC 50x470/110M, Czylok Sp. Z o.o., Jastrzębie Zdrój, Poland). The process was carried out for 1 h at a temperature of 800 °C, with a temperature increase of 10 °C per minute. The pyrolysis was carried out in a nitrogen atmosphere, where the gas flowed over the surface of the pyrolysed sludge samples. In the second step, the sludge after the pyrolysis process was subjected to chemical activation using NaOH (POCH, Gliwice, Poland). The sludge bath was carried out at a mass ratio of NaOH to sludge of 1:1 for 24 h. Then, after the chemical activation process, the sludge was washed with distilled water to remove the remaining reagents. The activated biochar prepared in this manner was utilised in a batch adsorption process for three synthetic dyes: AR 18, AG 16, and RB 81.

The structure of the produced activated biochar was characterised by measuring its porosity, specific surface area, and the type of functional groups present on its surface using Fourier-Transform Infrared Spectroscopy (FTIR). Additionally, microscopic images were captured using a stereoscopic microscope (Delta Optical, Nowe Osiny, Poland) and a biological microscope (Opta-Tech, Warszawa, Poland).

The FTIR measurement was conducted using a Jasco FTIR 6200 spectrophotometer coupled with the ATR Pro One View accessory (JASCO International Co., Ltd., Tokyo, Japan) to determine the functional groups present on the surface of the biochar. The specific surface area was measured using the N_2_ adsorption at 77 K method (Micromeritics ASAP 2020, Norcross, GA, USA). The methodology for measuring FTIR is the same as described in the papers by Pieczykolan and Krzyżowska [87], and Pieczykolan and Solecka [88]. For FTIR measurement, the study was conducted using a Fourier-transform infrared spectroscope in Attenuated Total Reflection mode, allowing for the measurement of the transmittance spectrum as a function of wavenumber in the range of 400–3500 cm^−1^. For the determination of specific surface area, the activated biochar was first dried at 105 °C, and then the sample was degassed at 300 °C for 6 h. Next, the adsorbent was subjected to N_2_ adsorption at 77 K using a Micromeritics ASAP 2020 from the USA (Norcross, GA, USA).

### 3.2. Dye Characteristics

Three different synthetic dyes were used in the study: Acid Red 18 (Boruta-Zachem Kolor S.A., Bydgoszcz, Poland), Acid Green 16 (Boruta-Zachem Kolor S.A., Bydgoszcz, Poland), and Reactive Blue 81 (Boruta-Zachem Kolor S.A., Bydgoszcz, Poland). Their characteristics are presented in Table 7.

Dyes AR 18 and RB 81 belong to the group of azo dyes. The characteristic occurrence of azo groups of the type =N-N-, -N=N- is inside their molecules. In contrast, AG 16 dye belongs to the triarylmethane class of dyes. The presence of a triphenylmethane structure inside the molecule is characteristic.

### 3.3. Batch Adsorption Process

The adsorption studies comprised four main stages: determining the effect of pH and contact time of activated biochar with dyes (present in aqueous solutions) on adsorption efficiency, examining the adsorption kinetics, and evaluating the adsorption isotherm. The experiments were conducted at a temperature of 293 K. All analyses were performed in triplicate, and the results are the arithmetic means.

#### 3.3.1. Effect of pH

In the first stage of the study, the impact of the pH value of the adsorption conditions was checked. For this purpose, for five different pH values of solutions (which were determined using NaOH or H_2_SO_4_) equal to 2, 4, 6, 8, and 10, the adsorption process of dyes was carried out, the initial concentrations of which were each 100 mg/dm^3^, and the contact time of the dye with activated biochar was 30 min. The amount of activated biochar was 2 g/dm^3^. After the process, the sludge was separated from the solutions, and the concentration of the remaining dye was measured using the spectrophotometric method with a UV-1800 spectrophotometer (Shimadzu, Kyoto, Japan). For this purpose, a method was employed using standard curves prepared for each dye at wavelengths of λ = 506 nm, λ = 631 nm, and λ = 583 nm for AR 18, AG 16, and RB 81, respectively.

The effectiveness of the adsorption process was determined based on the calculated amount of adsorbed dye, as per Formula (14).

(14) $$q_{e}=\frac{{(C}_{0}-C_{e})}{m_{sl}}$$

where:

*q_e_*—the amount of adsorbed dye per unit mass of biochar [mg/g];

*C_e_*—the dye concentration after adsorption process [mg/dm^3^];

*C*_0_—the initial dye concentration in aqueous solution [mg/dm^3^];

*m_sl_*—amount of biochar (sludge) [g/dm^3^].

#### 3.3.2. Effect of Contact Time

In the second stage of the study, tests were conducted using two different initial dye concentrations, 100 mg/dm^3^ and 700 mg/dm^3^, to assess the effect of contact time on the removal efficiency of individual dyes. For this purpose, for the pH value of dye solutions selected in the first stage, the adsorption process was carried out, where the amount of activated biochar was 2 g/dm^3^, and with the use of contact times of 5, 10, 15, 30, 45, 60, 90, 120, 150, 180 and 240 min. The dye concentration and adsorption efficiency were determined in a manner analogous to the first stage of the study.

#### 3.3.3. Adsorption Kinetics

An analysis of experimental data obtained in the stage of assessing the effect of activated biochar contact time on the effectiveness of adsorption was carried out, the goals of which were to analyse the fit of three kinetics models: pseudo-first-order (Equation (1)) [47], pseudo-second-order (Equation (2)) [48], and Elovich (Equation (3)) [49,50]. The kinetics model formulae are summarised in Table 1. The results of these analyses allowed for examining the type of adsorption process. The values of all three kinetics models were determined based on nonlinear estimation by minimising the root mean square error *RMSE* value (Equation (15)) using the Solver add-in of Microsoft Office 365.

Additionally, to analyse the mechanism of the adsorption process, an analysis of the intraparticle diffusion model was conducted [51,52]. The parameters of this model were determined based on linear estimation, based on the linear relationship expressed by Equation (6), and based on the *q_t_* versus *t*^0.5^ graph [92].

(15) $$RMSE=\sqrt{\frac{1}{n-2}\sum_{i=1}^{n}(q_{e,exp}-q_{e,calc}{)}_{i}^{2}}$$

where:

*q_e,exp_*—the dye adsorbed per unit mass of activated biochar, experimental data [mg/g];

*q_e,calc_*—the dye adsorbed per unit mass of activated biochar, estimated data [mg/g].

#### 3.3.4. Adsorption Isotherm

The last stage of the study was the experimental determination of adsorption isotherms. For this purpose, under established process conditions (determined adsorption pH and contact time), adsorption experiments were carried out using constant amounts of activated biochar and increasing concentrations of the dyes tested, ranging from 100 mg/dm^3^ to 1400 mg/dm^3^. The concentration of dyes after the process and the value of adsorbed charges were determined and calculated in the same way as in the previous stages of the study.

Moreover, based on the experimental results, an analysis of the fit of selected two- and three-parameter adsorption isotherm models was performed. The formulae of the analysed isotherm models are presented in Table 4. This analysis was conducted using nonlinear estimation by minimising the *RMSE* values (Equation (15)), utilising the Solver add-in in Microsoft Excel Office 365. The *R*^2^ value indicated the model’s fit to the experimental results.

## 4. Conclusions

The activated biochar was produced from sludge formed during water purification by means of the coagulation/flocculation process using aluminium salts. The adsorbent was characterised by porosity and specific surface area similar, and, in some cases, even greater, to the types of biochars produced from other waste materials. The conducted batch adsorption process studies of three dyes AR 18, AG 16, and RB 81 showed that, in all cases, it was necessary to use strongly acidic reaction conditions for the adsorption process to be effective. The results of the adsorption kinetics studies indicate the occurrence of a chemical adsorption process, as evidenced by the best fit to the experimental results obtained for the pseudo-second-order and Elovich models. Moreover, the analysis of the intraparticle diffusion model suggests that inner (intraparticle) diffusion is not the sole step controlling the rate of the process, as the value of *C* is different from 0. Therefore, mass transfer is also essential for the rate of the adsorption process in this case. Adsorption isotherm studies showed that, in the case of AR 18 and RB 81, the best fit to the experimental results was obtained for the SIPS model, which describes the adsorption process on a heterogeneous surface. In the case of AG 16, the best fit was obtained for the Jovanovic isotherm model, which describes the localised monolayer adsorption process without lateral interaction.

## Figures and Tables

**Table 1:** The models of kinetics were analysed in the study.

Kinetics Model	Formula	Eq.	Ref.	Parameters
Pseudo-first-order	$q_{t}=q_{e}\cdot \left(1-\exp\left(-k_{1}\cdot t\right)\right)$	(1)	[47]	*q_t_*—the dye adsorbed per unit mass of activated biochar at each contact time [mg/g]; *q_e_*—the dye adsorbed per unit mass of activated biochar at the equilibrium state [mg/g]; *k*_1_—the constant rate of the pseudo-first-order model [1/min]; *t*—contact time [min]; *k*_2_—the constant rate of the pseudo-second-order model [g/(mg·min)]; *a*—regarded as the initial sorption rate [mg/(g·min)]; *b*—constant related to the extent of surface coverage; *K_IPD_*—the intraparticle diffusion rate constant [mg/(g·min^0.5^)]; *C*—a constant depicting the boundary-layer effects [mg/g];
Pseudo-second-order	$q_{t}=\frac{q_{e}^{2}\cdot k_{2}\cdot t}{1+q_{e}\cdot k_{2}\cdot t}$	(2)	[48]	
Elovich	$q_{t}=\frac{1}{b}\cdot ln(1+a\cdot b\cdot t)$	(3)	[49,50]	
Intraparticle diffusion	$q_{t}=K_{IPD}\cdot t^{0.5}+C$	(4)	[51,52]	

**Table 2:** The parameters of adsorption kinetics.

Kinetics Model	Initial Dye Concentration	Param.	Unit	Dye		
				AR 18	AG 16	RB 81
Pseudo-first-order	100 mg/dm^3^	*k*_1_	1/min	0.17673	0.07703	0.15205
		*q_e_*	mg/g	49.3	49.4	50.1
		*R* ^2^		0.940	0.988	0.922
	700 mg/dm^3^	*k*_1_	1/min	0.07319	0.02434	0.16076
		*q_e_*	mg/g	207.3	232.7	276.4
		*R* ^2^		0.802	0.979	0.742
Pseudo-second-order	100 mg/dm^3^	*k*_2_	g/(mg·min)	0.00734	0.00246	0.00389
		*q_e_*	mg/g	50.6	51.6	55.2
		*R* ^2^		0.984	0.963	0.843
	700 mg/dm^3^	*k*_2_	g/(mg·min)	0.00042	0.00010	0.00087
		*q_e_*	mg/g	231.0	279.4	294.2
		*R* ^2^		0.928	0.992	0.878
Elovich	100 mg/dm^3^	*a*	mg/(g·min)	562.47	19.74	25.55
		*b*	g/mg	0.177	0.107	0.085
		*R* ^2^		0.863	0.870	0.720
	700 mg/dm^3^	*a*	mg/(g·min)	81.05	11.53	2034.78
		*b*	g/mg	0.026	0.014	0.031
		*R* ^2^		0.996	0.990	0.933

**Table 3:** Parameters of the intraparticle diffusion model.

	RB 81				AG 16				AR 18			
	100 mg/dm^3^		700 mg/dm^3^		100 mg/dm^3^		700 mg/dm^3^		100 mg/dm^3^		700 mg/dm^3^	
	1st Stage	2nd Stage	1st Stage	2nd Stage	1st Stage	2nd Stage	1st Stage	2nd Stage	1st Stage	2nd Stage	1st Stage	2nd Stage
*K_IPD_*	12.217	0.9392	62.612	6.6381	8.1955	0.1611	21.849	8.4398	11.419	0.1566	35.668	8.2545
*C*	0	43.315	9.9839	211.75	0.2068	47.423	0	118.04	2.0082	48.448	5.2061	118.13
*R* ^2^	0.9843	0.846	0.9746	0.9184	0.9971	0.6659	0.9873	0.9923	0.9700	0.5707	0.9793	0.9576

**Table 4:** The isotherm models analysed in the study.

Isotherm Model	Formula	Eq.	Ref.
Freundlich	qe=KF⋅Ce1n	(5)	[67]
Langmuir	$q_{e}=\frac{q_{m}\cdot K_{L}\cdot C_{e}}{1+K_{L}\cdot C_{e}}$	(6)	[68,69]
Jovanovic	$q_{e}=q_{max}\cdot \left[1-exp\left(-K_{J}\cdot C_{e}\right)\right]$	(7)	[70]
Dubinin–Radushkevich (D–R)	$q_{e}=Q_{s}\cdot \exp\left(-K_{DR}\cdot \varepsilon^{2}\right)$	(8)	[71]
	$\varepsilon =RT\cdot \ln\left(1+\frac{1}{C_{e}}\right)$	(9)	
	$E=\frac{1}{\sqrt{2\cdot K_{DR}}}$	(10)	
SIPS	$q_{e}=\frac{q_{mS}\cdot K_{S}\cdot C_{e}^{SP}}{1+K_{S}\cdot C_{e}^{SP}}$	(11)	[72]
Temkin	$q_{e}=B_{T}\ln\left(K_{T}\cdot C_{e}\right)$	(12)	[73]
	$B_{T}=\frac{R\cdot T}{b_{T}}$	(13)	

where: *K_F_*—constant associated to the relative adsorption capacity; *n*—exponent in Freundlich model; *q_m_*—the maximum adsorption capacity at monolayer coverage; *K_L_*—a Langmuir constant related to free energy of adsorption; *K_J_*—a constant related to the adsorption energy; *q_max_*—the maximum adsorption capacity; *Q_s_* is theoretical saturation capacity; *K_DR_*—a constant related to the adsorption energy; *ε*—Polanyi potential; *E*—mean free energy; *R*—gas constant (8.314 J/mol K); *T*—absolute temperature (K); *K_S_*—a constant related with energy of sorption and affinity of the system; *SP*—the exponent in SIPS model; *q_mS_*—the maximum monolayer adsorption capacity; *K_T_*—binding constant at equilibrium in Temkin model; *B_T_*—a parameter related to the heat of adsorption.

**Table 5:** The parameters of adsorption isotherms.

Isotherm Model	Parameter	AR 18	AG 16	RB 81
Freundlich	1/*n*	0.18115	0.31021	0.23449
	*K_F_*	65.001	23.004	52.970
	*R* ^2^	0.970	0.860	0.941
	*RMSE*	10.39	18.83	22.21
Langmuir	*q_m_*	202.07	204.49	253.54
	*K_L_*	0.05928	0.00845	0.01946
	*R* ^2^	0.848	0.911	0.935
	*RMSE*	29.71	47.46	26.66
Jovanovic	*q_max_*	197.00	173.86	233.00
	*K_J_*	0.02948	0.00726	0.01173
	*R* ^2^	0.913	0.994	0.924
	*RMSE*	37.02	6.80	32.72
Dubinin–Radushkevich	*Q_S_*	197.31	171.09	223.01
	*K_DR_*	212.006	1130.504	320.750
	*E*	0.05	0.02	0.04
	*R* ^2^	0.815	0.956	0.739
	*RMSE*	43.95	14.35	44.75
SIPS	*q_mS_*	1594.95	204.49	402.83
	*K_S_*	0.04151	0.00845	0.08526
	*SP*	0.200	1	0.442
	*R* ^2^	0.971	0.967	0.952
	*RMSE*	10.34	9.14	21.25
Temkin	*K_T_*	17.036	1.941	240.798
	*b_T_*	112.24	84.71	136.11
	*B_T_*	21.70	28.76	17.90
	*R* ^2^	0.931	0.889	0.760
	*RMSE*	15.89	21.70	35.90

**Table 6:** Examples of adsorption capacity for different biochar (adsorbent and activated carbon) derived from wastes.

Dye	Type of Dye	Adsorbent	Adsorption Capacity [mg/g]	Ref.
Reactive Black 5	Azo dye	macadamia-nut-residue-derived biochar	2.8–3.1	[39]
Reactive Orange 16	Azo dye	activated carbon derived from the stems of *Phyllanthus reticulatus*	67.93–100.5	[41]
New Coccine	Azo dye	protein-modified rice husk biochar	16–32	[40]
Reactive Black 5	Azo dye	pine tree logging residues	1260–1363	[43]
Remazol Brilliant Violet 5R	Azo dye	melunak-based activated carbon	238.33	[45]
Remazol Brilliant Violet 5R	Azo dye	rubberwood-sawdust-based activated carbon	204.08	[45]

**Table 7:** Dye characteristics [14,15,16,89,90,91].

	AR 18	AG 16	RB 81
Molecular structure	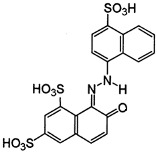	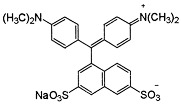	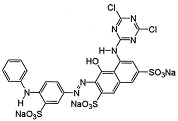
Molecular weight	604.48	560.62	808.49
Molecular formula	C_20_H_11_N_2_Na_3_O_10_S_3_	C_27_H_25_N_2_NaO_6_S_2_	C_25_H_14_Cl_2_N_7_Na_3_O_10_S_3_
C.I. number	16255	44025	18245
Colour	Red powder (red)	Green (variegated dark green powder)	Blue powder (red light blue)
Dye class	Single azo class	triarylmethane class	Single azo class
Topological polar Surface area	242 Å^2^	137 Å^2^	304 Å^2^

## Data Availability

The data are available upon request due to privacy concerns.
